# A Fast and Low-Power Detection System for the Missing Pin Chip Based on YOLOv4-Tiny Algorithm

**DOI:** 10.3390/s23083918

**Published:** 2023-04-12

**Authors:** Shiyi Chen, Wugang Lai, Junjie Ye, Yingjie Ma

**Affiliations:** 1 School of Mechanical and Electrical Engineering, Chengdu University of Technology, Chengdu 610059, China; 2 College of Computer Science and Cyber Security (Oxford Brookes College), Chengdu University of Technology, Chengdu 610059, China

**Keywords:** chip detection, FPGA, inference accelerator, low-power system

## Abstract

In the current chip quality detection industry, detecting missing pins in chips is a critical task, but current methods often rely on inefficient manual screening or machine vision algorithms deployed in power-hungry computers that can only identify one chip at a time. To address this issue, we propose a fast and low-power multi-object detection system based on the YOLOv4-tiny algorithm and a small-size AXU2CGB platform that utilizes a low-power FPGA for hardware acceleration. By adopting loop tiling to cache feature map blocks, designing an FPGA accelerator structure with two-layer ping-pong optimization as well as multiplex parallel convolution kernels, enhancing the dataset, and optimizing network parameters, we achieve a 0.468 s per-image detection speed, 3.52 W power consumption, 89.33% mean average precision (mAP), and 100% missing pin recognition rate regardless of the number of missing pins. Our system reduces detection time by 73.27% and power consumption by 23.08% compared to a CPU, while delivering a more balanced boost in performance compared to other solutions.

## 1. Introduction

In the field of industrial chip production, defects often occur due to various factors, such as technological processes, materials, and environment. Defect detection is a critical part of the chip production process to ensure high-quality and reliable products [1]. However, traditional manual detection methods are inefficient and unable to meet the growing demand for high-speed and accurate detection in modern chip production [2] and are gradually being replaced by automatic detection technology. In traditional machine vision detection, the automatic optical inspection method has gained popularity for quality detection of PCBs in electronic devices using optical sensors and computer technology [3,4]. However, this approach still presents urgent problems with the measurement of key geometric dimensions of chip pins [5,6]. To address this challenge, various methods have been proposed for efficient defect detection. For instance, Liu et al. proposed an adaptive threshold FAST feature points extraction for chip pin feature extraction and used cascaded region segmentation to determine the pin positions, improving the efficiency of defect detection for missing, bent, and bonded chip pins using machine vision [7]. Qiao et al. used a computer terminal to control an industrial camera and processed it using Halcon software algorithms to monitor chips with surface scratches in real time [8]. Lu et al. used a binocular vision inspection system, combined with a corner detection algorithm and a preprocessing algorithm of gradient correlation matrices, to automatically identify the chips on the conveyor belt [9]. As chips become increasingly integrated and complex, defects produced in industrial production are often diverse in type, complex in features, and variable in background. Traditional machine vision technology is inefficient and ineffective in extracting defect features sufficiently and effectively and has become inadequate for the task. In recent years, deep learning models, especially convolutional neural networks (CNNs), have shown superior performance and adaptability in defect detection, making them particularly applicable to various industrial scenarios [10,11,12,13,14]. Ding et al. combined feature pyramidal convolutional networks with the Faster R-CNN for PCB surface defect detection [15]. Yang et al. fine-tuned the YOLOv3 network for multiple classes of chip defect detection [16]. Ghosh et al. proposed a CNN-based classification scheme and two unsupervised techniques to identify bent and corroded pins from RGB maps and depth maps [17]. Hou et al. deployed some lightweight networks such as AlexNet and GoogLeNet using a single-board computer to achieve edge device detection of laser chip defects [18].

However, the current method of deploying machine vision in industrial scenarios still involves using power-hungry computers as terminal devices. While solutions related to using neural networks for chip defect detection have been proposed, they mostly focus on algorithmic improvements and do not provide specific industrial deployment strategies. Additionally, the machine vision solutions used by previous researchers can only detect one chip at a time, which significantly reduces detection efficiency.

To address these issues, this paper proposes an innovative approach that utilizes the YOLOv4-tiny algorithm with multi-object detection capability [19] and deploys it on an FPGA system to detect missing pins in chip production. The FPGA platform offers significant advantages, including low power consumption, small size, high performance, and flexible sensor integration [20,21,22,23,24], making it an ideal choice for industrial deployment.

In this paper, we propose an innovative design of an FPGA accelerator structure with two-layer ping-pong optimization and multiplex parallel convolution kernels, resulting in a more balanced improvement among precision, inference time, and power consumption compared to previous solutions. Furthermore, our system addresses the limitations of previous chip detection methods, such as the inability to simultaneously detect multiple chips and the neglect of continuous missing pins on a chip. As a result, our system is capable of coping with a wider range of complex industrial environments.

Overall, this paper demonstrates the tremendous potential of combining advanced algorithms with innovative hardware design to achieve efficient and reliable defect detection in industrial scenarios. Our method offers an effective solution for detecting missing pin defects in industrial chip production. By utilizing FPGA technology, we not only improve the performance of the system but also offer a feasible deployment strategy for industrial applications.

## 2. Experimental Setup

In this section, we first introduce our experimental platform and provide specific hardware information. Then, we separately describe the development tools used in this study for both the neural network training process and the hardware development process.

### 2.1. Experimental Platform

**Figure 1:**
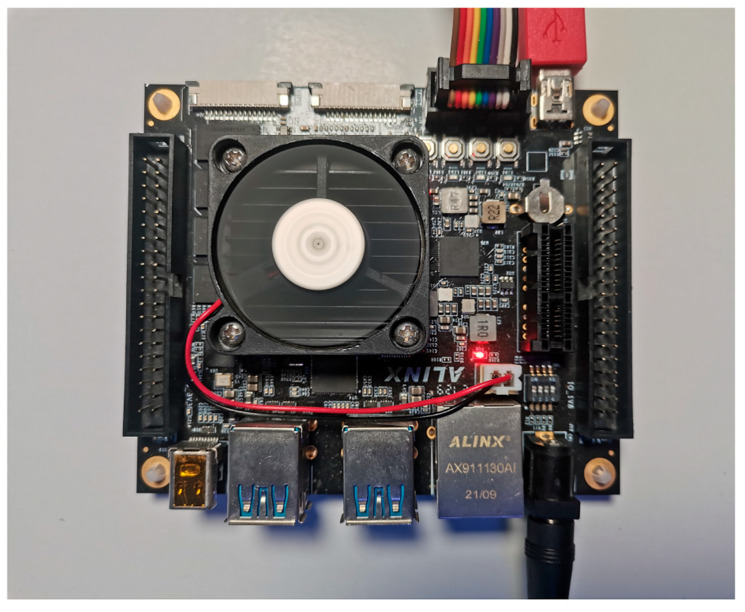
The AXU2CGB development board.

To meet industrial requirements and keep costs manageable, we chose the AXU2CEG development board, which measures only 10.00 cm × 8.50 cm, as shown in Figure 1. It features an ARM Cortex-A53 processor for handling complex tasks and future expandability, as well as an ARM Cortex-R5 coprocessor to improve real-time processing efficiency. The AXU2CGB supports multiple interfaces, including USB, HDMI, Ethernet, SPI, and UART, among others, making it suitable for industrial inspection scenarios. The AXU2CEG is cost-effective due to its low-end FPGA from Xilinx’s Zynq UltraScale+ MPSoC family, which has limited internal resources. Therefore, the design can easily expand to simultaneously detect more images by switching to a more powerful FPGA chip.

### 2.2. Development Tools

In the neural network training process, we first perform image preprocessing and other image-related input/output operations using the OpenCV 4.6.0 computer vision library. Then, we construct the YOLOv4-tiny network using the PyTorch 1.11.0 deep learning framework and its associated Torchvision 0.12.0 library for framework construction. Finally, we employ various third-party libraries for training. To evaluate the training results, we use the Matplotlib 3.4.3 visualization library to plot the Epoch-Loss and Epoch-mAP graphs.

In the hardware development process, we use three development tools: Vivado HLS, Vivado, and Xilinx Vitis IDE, all with version numbers of 2019.2. First, we design the bottom-level convolution IP in Vivado HLS. Then, we construct the system hardware platform in Vivado and obtain resource and power consumption information. Finally, we construct the top-level YOLOv4-tiny network based on this hardware platform in the Xilinx Vitis IDE and analyze inference time and precision.

## 3. Theoretical Analysis

### 3.1. YOLOv4-Tiny Network

YOLO is an object detection model proposed to meet object detection tasks in different situations. Its principle is to learn object positions directly from object types in a lightweight network. Essentially, YOLO is to directly locate and label objects by learning them once, allowing it to quickly achieve good performance in the overall image [25]. Therefore, a series of versions of YOLO networks have been widely used in the industry. For example, Adibhatla et al. applied YOLOv2-tiny to the detection of printed circuit board defects [26], and Wang et al. applied YOLOv3 to the detection of wearing safety helmets [27].

In this paper, the 416 × 416 YOLOv4-tiny network is used. Compared to the almost 60 million parameters of YOLOv4, YOLOv4-tiny is only one-tenth of it, which not only makes its detection speed six to eight times faster than YOLOv4 [28], but also occupies a small amount of storage space. Apart from that, the relatively simple structure of YOLOv4-tiny also enables faster inference speed on devices with limited computing resources. On the other hand, the higher versions of the YOLO model introduce more new features, such as attention mechanisms, which increase the complexity. Although YOLOv4-tiny may have slightly lower accuracy than its higher versions, it still performs well and can meet many practical needs. Therefore, it is more suitable for deployment on embedded devices such as FPGAs.

**Figure 2:**
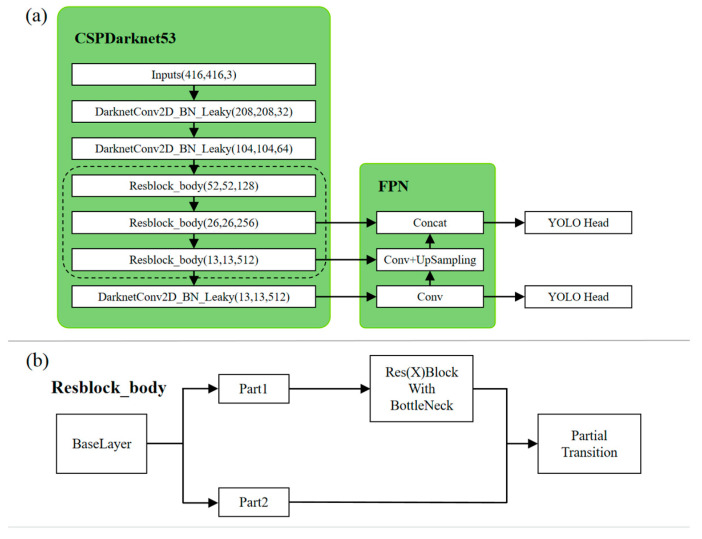
(**a**) YOLOv4-tiny network structure. (**b**) Resblock_body module structure.

The YOLOv4-tiny network structure has 38 layers and uses three residual units: a Leaky ReLU for the activation function, two feature layers for target classification and regression, and a Feature Pyramid Network (FPN) to merge the effective feature layer. The YOLOv4-tiny network structure is shown in Figure 2a.

The CSPDarknet53 module in Figure 2a completes three operations in turn: DarknetConv2D, Batch Normalization (BN), and the Leaky ReLU activation function, which increases the learning capabilities of the CNN and ensures accuracy while reducing memory costs. The Resblock_body module is then used to prevent problems such as the vanishing gradient problem.

In Figure 2b, the ResBlock_body module divides the Base Layer into two parts, Part1 and Part2. Part2 is simply a copy of the input data. Part1, on the other hand, needs to undergo a residual network operation and is subsequently combined with Part2 through a fusion process to obtain the Partial Transition. This process is a cooperative task performed by Part1 and Part2 working together and can be seen as two fusions of residual features.

The main operation of the FPN is to upsample the higher layer features of the two feature layers twice, while the lower layer features undergo 1×1 convolution to change the number of channels of the lower layer features. Then, the corresponding upsampled results from the previous higher layers are added to the results of the 1×1 convolution.

After the above operation, two YOLO Head feature output layers of sizes (26, 26, 255) and (13, 13, 255) are obtained. Both YOLO Head feature output layers contain the width, height, and number of channels of the predicted result, which are then decoded by YOLO to show the position of the entire prediction frame.

### 3.2. Fixed-Point 16-Bit Quantization

CNNs are robust to data precision and can reduce computational resources by reducing data bit-width while keeping precision constant [29,30,31]. Moreover, as the data bit width is reduced, the amount of data transferred is also reduced. Before quantization, the data is in 32-bit floating point, and YOLOv4-tiny contains approximately 5.9 MB of parameters [32], which would require 23.6 MB of storage, and more advanced networks tend to contain a larger number of parameters. Therefore, this paper uses fixed-point 16-bit quantization for the model’s weights and bias parameters, input and output feature maps, and intermediate results. Due to the limited resources of this development board and to reduce computational effort, we set the number of decimal places to nine. Fixed-point data $x_{fixed}$ is represented as a complement, and its representation and conversion relationship with floating-point data $x_{float}$ are as follows:

(1) $$x_{fixed}=\sum_{i=0}^{15}B_{k}\times 2^{-9}\times 2^{k},\;B_{k}\in \left\{0,1\right\}\;,$$

(2) $$x_{fixed}=\left(int\right)\left(x_{float}\times 2^{9}\right)\;,$$

(3) $$x_{float}=\left(float\right)x_{fixed}/2^{9}\;,$$

where $\;B_{k}$ is the kth bit of the 16-bit fixed-point data.

### 3.3. Fusion of the Batch Normalization (BN) Layer and Convolution Layer 

In general, the BN layer is located after the convolution layer and normalizes the feature map, an operation that speeds up the network learning rate and has the effect of regularization. By fusing the BN layer to the upper convolutional layer, the two-step operation can be reduced to one step, which has an accelerated effect.

In the convolution layer, the main convolution process is that the filter traverses the input image through a set number of steps. The equation for this operation is as follows:

(4) $$y_{conv}=\omega \cdot x+b\;,$$

where the convolution kernel weight ω and bias b is used to perform a linear operation on an element x in its input feature map and $y_{conv}$ is the result of the convolution calculation.

In the BN layer, it is necessary to calculate the mean and variance of a minibatch of elements, then subtract the mean by x and divide the standard deviation, and finally perform the affine operation. The specific calculation formula is as follows:

(5) $$\mu_{\beta }=\frac{1}{m}\sum_{i=1}^{m}x_{i}\;,$$

(6) $$\delta_{\beta }^{2}=\frac{1}{m}\overset{m}{\underset{i=1}{\sum }}{\left(x_{i}-\mu_{\beta }\right)}^{2}\;,$$

(7) $$\overset{\wedge }{x_{i}}=\frac{x_{i}-\mu_{\beta }}{\sqrt{\sigma_{\beta }^{2}+\varepsilon }}\;.$$

where m is the number of elements in a minibatch, $x_{i}$ is the ith element in a minibatch, and $\mu_{\beta }$ and $\delta_{\beta }^{2}$ are the mean and variance of a minibatch of elements, respectively. ε is a small constant set to avoid division by zero errors. $\overset{\wedge }{x_{i}}$ is the result after normalizing $x_{i}$.

By introducing the zoom variable γ and the translation variable β obtained by model training for affine operation, we can obtain the processed convolution formula:

(8) $$BN_{\gamma \beta }\left(x_{i}\right)=\gamma {\overset{\wedge }{x}}_{i}+\beta \;,$$

where $BN_{\gamma \beta }\left(x_{i}\right)\;$ is the result of the processed convolution calculation.

The final fusion step brings the convolution formula directly into the BN formula, e.g.,

(9) $$BN_{\gamma \beta }\left(x_{i}\right)=\gamma \frac{\omega \cdot x+b-\mu_{\beta }}{\sqrt{\sigma_{\beta }{}^{2}+\varepsilon }}+\beta =\frac{\omega \cdot \gamma }{\sqrt{\sigma_{\beta }{}^{2}+\varepsilon }}\cdot x+\frac{\gamma }{\sqrt{\sigma_{\beta }{}^{2}+\varepsilon }}\cdot \left(b-\mu_{\beta }\right)+\beta \;,$$

(10) $$\mathrm{Order}\;\overset{\wedge }{\omega }=\frac{\omega \cdot \gamma }{\sqrt{\sigma_{\beta }{}^{2}+\varepsilon }}\;,$$

(11) $$\overset{\wedge }{b}=\frac{\gamma }{\sqrt{\sigma_{\beta }{}^{2}+\varepsilon }}\cdot \left(b-\mu_{\beta }\right)+\beta \;,$$

(12) $$\mathrm{Then}\;BN_{\gamma \beta }\left(x_{i}\right)=\overset{\wedge }{\omega }\cdot x+\overset{\wedge }{b}\;.$$

With the above transformation, the BN layer is fused to the convolutional layer, reducing the amount of model operations and random access memory.

### 3.4. Mean Average Precision (mAP) Derivation

In object detection, the precision of each category can be represented using a Precision-Recall curve, which is a graph of recall on the *x*-axis and precision on the *y*-axis. The curve reflects the trade-off between precision and recall in the detection algorithm. For each category, the average precision (AP) is calculated as the area under the Precision-Recall curve. The higher the AP, the better the performance of the object detection algorithm in that category.

mAP is a metric used to evaluate the performance of object detection models. It is the average of the AP scores for all object categories. Therefore, a higher mAP value indicates higher detection precision for the model across all categories. The formulas for precision (P), recall (R), and AP calculation are as follows:

(13) $$P=\frac{TP}{TP+FP},$$

(14) $$R=\frac{TP}{TP+FN},$$

(15) $$AP=\int_{0}^{1}P\left(R\right)dR,$$

(16) $$mAP=\frac{\sum_{i=1}^{N}AP}{N}.$$

where True Positives (TP) are the number of correctly detected objects, False Positives (FP) are the number of incorrectly detected objects, False Negatives (FN) are the number of objects not detected by the model, and N is the number of categories.

## 4. Methods

In this section, we first present the system workflow and hardware architecture and the YOLOv4-tiny network in a holistic manner, then show the scheme for dataset enhancement, and finally propose a two-layer ping-pong optimized FPGA accelerator architecture for FPGA deployment, in which the design of the convolution kernel is described in detail.

### 4.1. System Model

#### 4.1.1. System Workflow

**Figure 3:**
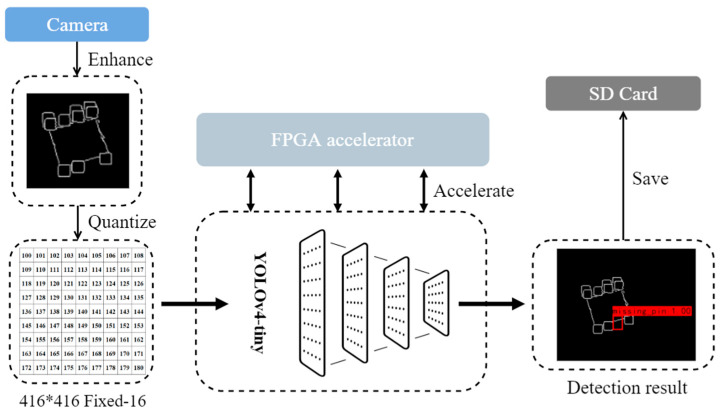
Chip defect detection system workflow.

The detection system uses an accelerated deep learning algorithm to screen the missing pin chip and locate the missing pin locations by detecting chip photos on the process line. First, the chip photos taken by the camera are edge-extracted for enhancement, and then 16-bit fixed-point quantization is performed. Quantized images and BN fused weights are fed into a YOLOv4-tiny network, where the FPGA accelerator performs acceleration operations to produce inference results. If a defect is detected, it is stored on the SD Card; otherwise, it continues to read the next image. The system workflow is shown in Figure 3. 

#### 4.1.2. System Hardware Architecture

**Figure 4:**
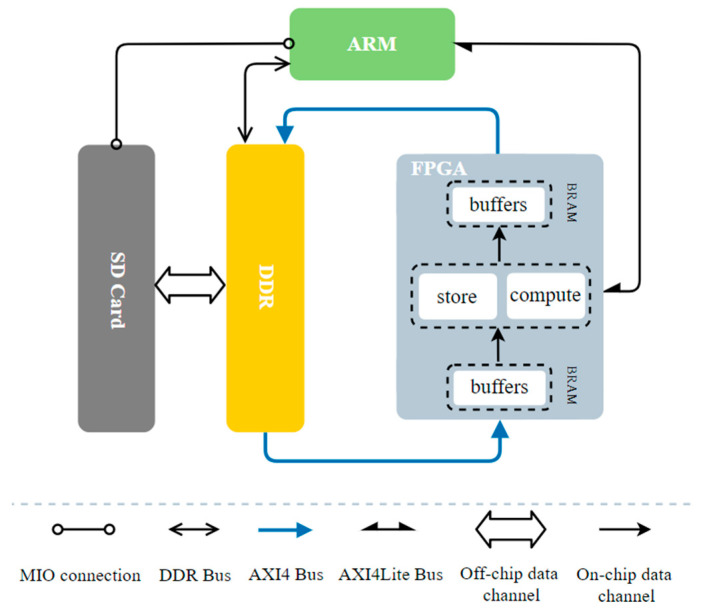
Hardware architecture of the chip defect detection system.

The storage component of the chip defect detection system comprises an SD Card, DDR, and on-chip storage resources, specifically Block RAM (BRAM). The overall system architecture is illustrated in Figure 4. Due to the relatively simple nature of the detection task in this project, only one Cortex-A53 processor is utilized without any operating system being employed.

The ARM processor is responsible for executing the application program, which controls the entire system. This includes constructing the overall architecture of the YOLOv4-Tiny network, preprocessing the image, weight, and bias files stored on the SD Card, loading them into DDR, continuously calling the convolutional accelerator in the FPGA, and storing the inference results back to the SD Card.

On the other hand, for specific hardware acceleration and customized design, the FPGA generates IP cores through Vivado HLS. Its functions include loading data from DDR into respective BRAM buffers in a multi-channel parallel transmission manner, performing convolutional acceleration operations in the compute and store modules in a two-layer ping-pong form, and finally storing the results back into DDR.

The blue line in Figure 4 is controlled by the FPGA and is the high-speed bus of the AXI4 bus, which adopts a multiplex burst transmission mode and can improve transmission efficiency and increase throughput [33,34,35]. It allows a large amount of data interaction between the off-chip DDR and the on-chip BRAM. The remaining black lines are controlled by ARM, which transmits commands and configurations to the accelerator IP via the AXI4Lite bus and sends read and write signals to the DDR and SD Card via the DDR bus and multiuse I/O (MIO), respectively.

### 4.2. Dataset Enhancement

Due to the lack of large-scale open-source datasets for chip defect detection, we need to create our own dataset. This approach allows us to design and collect data tailored to the specific issues we are studying and adjust the dataset’s size and content as needed. Additionally, because there are so many types of chip packages, we only select the SOP series, a typical package series, in order to better control the complexity of the experiments and facilitate comparison and validation of results. 

**Figure 5:**
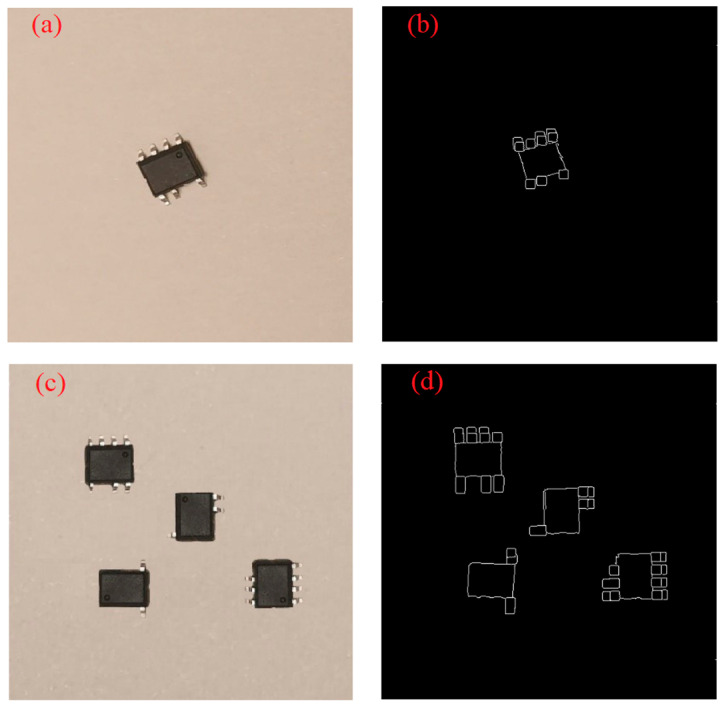
Chip data set. (**a**) One chip before enhancement. (**b**) One chip after enhancement. (**c**) Multiple chips before enhancement. (**d**) Multiple chips after enhancement.

Before feeding into the inference, we first perform image enhancement by binarizing the images and using the Canny algorithm for edge extraction, which simplifies redundant features such as image color and emphasizes the feature of missing pins or not. The corresponding target detection model trained by this operation was tested and proved to perform better. The image enhancement process before and after is shown in Figure 5. Considering that industrial deployment requires high efficiency in the production line, we add multiple chips to the field of view to enhance the batch processing capability of the detection system. 

### 4.3. FPGA Deployment

#### 4.3.1. Overall Design of the FPGA Accelerator

**Figure 6:**
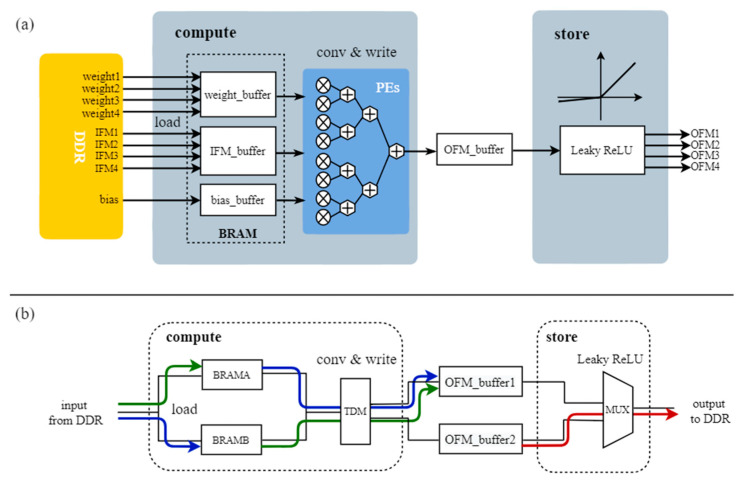
(**a**) Internal architecture of the FPGA accelerator. (**b**) The two-layer ping-pong design.

The FPGA accelerator consists of two modules: compute and store; the overall design of the FPGA accelerator is shown in Figure 6. In the compute module, the parameters are loaded into the respective buffers in BRAM in the form of multiplex parallel burst transmission via the AXI4 high-speed bus, and convolution is realized by the processing elements (PEs) in the FPGA through multiplication and addition operations. In the store module, the PEs perform Leaky ReLU activation and send the results out in multiplex parallel, as shown in Figure 6a. For the blue and green lines in Figure 6b, they represent operations that are completed at different times under fine-grained ping-pong, while the red line represents operations under coarse-grained ping-pong.

For the two-layer ping-pong operation, the accelerator first caches the DDR input to BRAMA and BRAMB, then performs ping-pong operations on load and convolve and write, and writes the result to one of the OFM_buffers in the form of time division multiplexing (TDM) to complete the compute module, as shown in the blue and green lines in Figure 6b. At the same time, we perform coarse-grained ping-pong between the compute module and the store module, i.e., while the compute module writes to OFM_buffer1, the result of the previous calculation cache is read out from OFM_buffer2 to DDR, as shown in the red line in Figure 6b. The work of the red line, blue line, and green line is carried out simultaneously, allowing fine-grained ping-pong within modules and coarse-grained ping-pong between modules to be performed at the same time. Thus, from the DDR side, data is sent out and in without interruption, and from the BRAM side, the utilization of on-chip resources is greatly improved.

#### 4.3.2. Detail Design of Convolution Kernel

Convolutional operations account for more than 90% of computation in most neural networks [36]. Therefore, accelerating the convolutional operations can significantly reduce the processing latency of the entire network. Since FPGA on-chip storage resources are very limited and the weights of the convolutional neural network and the feature maps of the intermediate computation results have large storage footprints, it is obviously impractical to cache the entire feature maps, so we adopt a loop tiling strategy. Suppose the input feature map I is of size N×R×C, the weight W is of size M×N×K×K, the output feature map O is of size M×R×C, and the block factors for the input channel, output channel, and output feature map height and width are Tn, Tm, Tr, Tc. After each block is computed, the next block of input features and weights is then read for computation.

This design uses parallel input channels and parallel output channels. That is, convolutions of feature maps and weights for multiplex input channels are computed simultaneously, and partial results and multiplex output feature maps are computed simultaneously. The corresponding pseudo code is shown in Algorithm 1.
**Algorithm 1** Pseudo code of optimized convolutional kernels for (r = 0; r < R; r += Tr) {//Traverse the rows of the feature map in steps of *Tr*  for (c = 0; c < C; c += Tc) {//Traverse the columns of the feature map in steps of *Tm*   for (m = 0; m < M; m += Tm) {//Traverse the channels of the output feature map in steps of *Tm*    for (n = 0; n < N; n += Tn) {//Traverse the channels of the input feature map in steps of *Tn*     load I [n:n + Tn][r:r + Tr + K][c:c + Tc + K] to IFM_buffer;     load W [m:m + Tm][n:n + Tn][:][:] to weight_buffer; *    //Compute partial sum and write to OFM_buffer*    for (ii = 0; ii < K; ii++)//Traverse the rows of the convolution kernel     for (jj = 0; jj < K; jj++)//Traverse the columns of the convolution kernel      for (rr = 0; rr < Tr; rr++)//Traverse the rows of the feature map block       for (cc = 0; cc < Tc; cc++)//Traverse the columns of the feature map block #pragma HLS PIPELINE II = 1        for (mm = 0; mm < Tm; mm++)//Traverse the channels of the output feature map block         for (nn = 0; nn < Tn; nn++)//Traverse the channels of the input feature map block          OFM_buffer [mm][rr][cc]+=           IFM_buffer [nn][rr + ii][cc + jj] × weight_buffer [mm][nn][ii][jj];      store OFM_buffer to O [m:m + Tm][r:r + Tr][c:c + Tc];      }}}}

We use PIPELINE optimization to unroll all its internal loops in parallel, and each iteration is completed by its own circuit, so there is no need for time division multiplexing of the common circuit. As a result, each iteration of the input and output channel loops is executed in parallel, and all iterations are completed in one cycle, resulting in an increase in computational speed.

## 5. Results

In this section, we first show the resource consumption of FPGA. Then, we show the model training process and results, and then, in order to verify the performance of our algorithm, we present the inference results on CPU and FPGA, respectively.

### 5.1. Resource Consumption

We present the overall resource consumption of the system and the resource consumption of convolutional operations that occupy the maximum computation in the system (as a percentage of total resources) in Table 1. Due to the limited FPGA resources of the AXU2CGB platform, our YOLOv4-tiny network almost exhausts the DSP units and consumes a substantial amount of LUT units. The importance of convolutional operations to the entire system can be seen from the convolutional consumption, which also suggests that optimizing the convolution module can greatly enhance the performance of the entire system.

### 5.2. Model Training and Results

**Figure 7:**
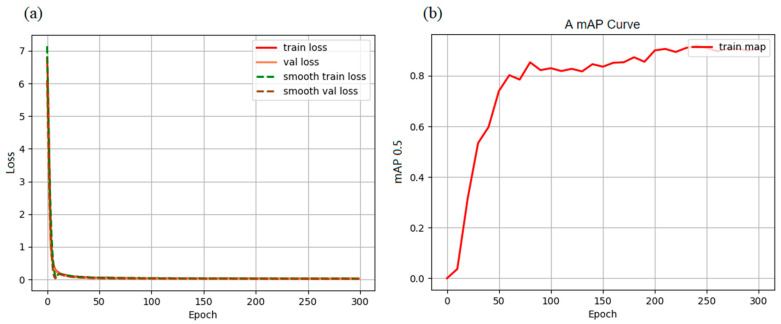
(**a**) Training Epoch-Loss diagram. (**b**) Training Epoch-mAP diagram.

In training the target detection model, we use 505 images for training the network, 49 images for evaluation during the training process, and 50 images for testing the generalization effect of the model. Because the dataset is small, 50 images from the validation set are combined into the training set. The maximum learning rate is set at 0.001, the minimum learning rate is set at 0.0001, and the total number of epochs is set at 300. During the training process, the algorithm generates various metrics, such as loss and mAP, which are collected at the end of each training cycle. Typically, a lower loss value and higher mAP indicate better predictive performance of the model. The loss and mAP with the number of epochs are shown in Figure 7.

It can be seen from Figure 7a that the loss value of training decreases rapidly in the first 10 epochs, falling below 0.1 in the 25th epoch, and that the decreasing trend tends to be stable, and the final loss drops to about 0.023. In Figure 7b, the mAP increases rapidly in the first 60 epochs and continues to increase slowly thereafter, reaching a maximum of approximately 0.92. Therefore, it can be seen that the operation achieves a good training effect.

### 5.3. Inference Results

**Figure 8:**
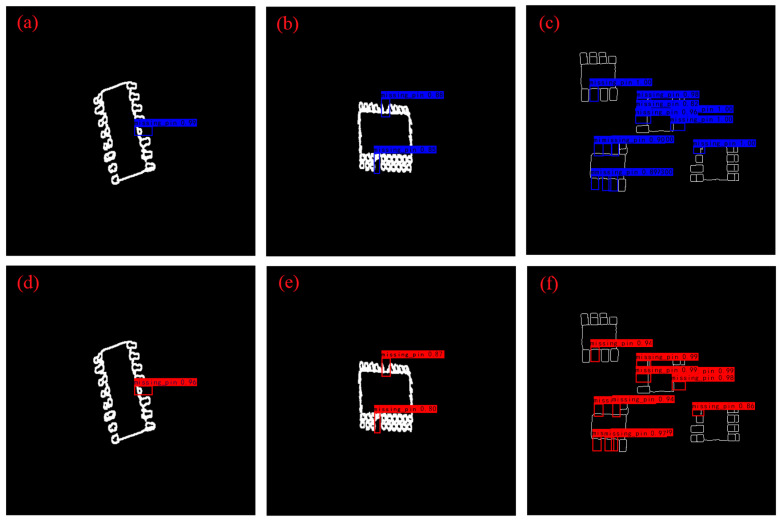
Inference results. (**a**) The result of the CPU inferring SOP16 chip. (**b**) The result of the CPU inferring SOP20 chip. (**c**) The result of the CPU inferring multiple SOP8 chips. (**d**) The result of the FPGA inferring SOP16 chip. (**e**) The result of the FPGA inferring SOP20 chip. (**f**) The result of the FPGA inferring multiple SOP8 chips.

We infer SOP8, SOP16, and SOP20 chips separately and compare the results obtained between the CPU and FPGA platforms, as shown in Figure 8. The blue recognition box represents the CPU inference result, the red recognition box represents the FPGA inference result, and the recognition box above displays the detected object type and confidence.

From Figure 8, we can see that the precision is relatively high. In comparison, the precision of FPGA inference is slightly lower than that of the CPU, and the recognition accuracy of SOP20 chips decreases due to the increase in the number of pins and the decrease in chip size. Additionally, our system has the ability to simultaneously detect multiple chips, as shown in Figure 8c,f. Due to the presence of multiple missing pins in the field of view, especially when a chip has multiple consecutively missing pins, the detection difficulty is high. Therefore, there are a few missing pins that are not detected in Figure 8c,f, which reduces the overall precision. However, considering the current industrial scenario, a chip will be treated as defective as long as there is a missing pin. Thus, if we do not care about the number of missing pins, just from the ability to distinguish between normal and defective chips, we achieve a recognition rate of 100% and can meet the requirements of general industrial deployments.

After testing was performed on the dataset, we obtained the following results: On the CPU platform, the detection time for each photo was 1.751 s, with a power consumption of 15.25 W and a precision of 90.76%. On the FPGA platform, the detection time for each photo was 0.468 s, with a power consumption of 3.52 W and a precision of 89.33%.

## 6. Comparison and Discussion

In this section, we compare our approach for detecting missing pin chips with other existing methods, highlighting their respective merits and downsides. We then explore the detection performance on both CPU and FPGA platforms and compare and discuss the detection time, precision, and power consumption of deploying neural networks on other platforms. Finally, we list some limitations and possible areas for development.

### 6.1. Comparison with Other Detection Solutions

To evaluate the effectiveness of our detection approach, we compared it to three other solutions commonly used in the industry:

Solution [7] utilizes machine vision to detect geometric differences in chip dimensions. Compared to our approach, this solution obtains higher precision and faster detection speeds, and it is also able to detect pin bending and bonding defects. However, its disadvantage lies in the complexity of the entire detection device, which makes it unsuitable for edge computing requirements due to its size and power consumption.

Solution [8] uses machine vision to screen defective chips through template matching. Compared to our approach, this solution provides a more intuitive human-computer interaction interface and achieves higher precision. However, its drawbacks include the use of a high-power computer as a terminal and the need for manual addition of each chip, making it unsuitable for industrial automation scenarios.

Solution [9] employs a binocular vision measurement system to detect pin defects by extracting corner features of chip pins. Compared to our approach, this solution provides a more comprehensive measurement system and can adapt to more complex lighting environments. However, its drawbacks include the use of a high-power computer as a terminal and the need to set different algorithms for each type of chip, making it more difficult to transplant.

Moreover, these three approaches cannot simultaneously detect multiple chips, nor do they consider the case of continuously missing pins on a chip. In comparison, our detection solution can cope with a wider range of complex industrial environments.

### 6.2. Comparison with Other Platforms Deploying Neural Networks

To further validate the performance of our algorithm on the FPGA platform, we also compare it with an unquantified CPU platform, as well as with the Zedboard [37,38], a platform running on a system on programmable chip (SOPC) consisting of ARM and FPGA, and the Stratix V GSD8 [39], a platform consisting solely of FPGA. The comparison is conducted in terms of inference time, mAP, power, and so on, and the results are presented in Table 2. These metrics are crucial for industrial applications as fast, low-power, and accurate detection of objects can impact efficiency and productivity.

From Table 2, it can be observed that the use of fixed-point 16-bit quantization leads to some precision loss compared to the original 32-bit floating-point data on the CPU. Nevertheless, the mAP of our accelerated IP decreases only by 1.43% compared to the original floating-point 32-bit model. Moreover, our system’s inference time, which is the average time taken to perform inference on each image in the test set, reduces by 73.27% compared to the unquantified CPU platform, and the power consumption is only 23.08% of the CPU.

Compared to paper [37], although our power consumption is higher, the detection time decreases significantly, and the mAP is also higher. Similarly, compared to paper [38], our power consumption is slightly higher, but the detection time is shorter, and the mAP increases significantly. Furthermore, compared to paper [39], our power consumption decreases significantly, the detection time is shorter, and the mAP is higher. Overall, our approach achieves a more balanced optimization in terms of detection precision, detection time, and power consumption, making it more suitable for industrial applications.

### 6.3. Limitations and Potential Applications

After comparison, we identify certain limitations in the application of our system:

- As neural networks grow more complex, richer FPGA resources are needed. To deploy a high-performing neural network on a resource-constrained FPGA, it is necessary to implement optimizations such as network compression and pruning to reduce the computational load and number of parameters.
- FPGA is not suitable for floating-point calculations, and quantization inevitably leads to reduced precision. A more fine-grained quantization scheme needs to be chosen to mitigate the impact of quantization on precision.
- This method primarily aims to detect chips in SOP packages. Since different chip packages may have distinct characteristics and specifications, the dataset requires extensive modification and augmentation, and the model must undergo rigorous training to ensure effective detection performance.

Based on the advantages and limitations of our system, we suggest several possible uses and influences on the industry:

- The system can be combined with advanced sensors, Internet of Things (IoT) technology, and human-machine interaction technology to expand its application prospects.
- The target detection technology can be applied to high-precision, low-power consumption-demanding fields such as intelligent security, traffic safety, and autonomous driving, significantly improving production efficiency and reducing costs.
- This method provides an effective solution for deploying neural networks on FPGA and offers new ideas for the application of FPGA technology in the electronics industry.

We will continue to explore these directions in future research to further improve the performance and applicability of the system.

## 7. Conclusions

This paper presents an FPGA-based missing pin chip detection system using the YOLOv4-tiny network that achieves fast and low-power operation through the implementation of various strategies, which are discussed in detail below:

1. To improve the precision and FPGA computational performance, we preprocess image and weight files before feeding them into the YOLOv4-tiny network, using image enhancement, fixed-point 16-bit quantization, and the fusion of the BN layer and convolution layer.
2. For the FPGA accelerator architecture, we design a two-layer ping-pong operation for uninterrupted read and write of off-chip memory DDR data. A loop tiling strategy is first used to cache feature blocks, and then the input and output channels are multiplexed in parallel to accelerate the convolution.
3. The final result shows a precision of 89.33% on the AXU2CEG development board, which takes 0.468 s to process a single photo, consuming 3.52 W. Compared to a CPU platform, the time spent is reduced by 73.27%, and the power consumption is reduced to 23.08%. Moreover, the system can support multi-object detection scenarios, which greatly improves the detection efficiency.

Overall, this system addresses the gap in the field of efficient and low-power multi-object detection for detecting missing pin chips and achieves a more balanced boost in performance compared to other solutions, which meets the expected target.

## Figures and Tables

**Table 1:** Resource consumption of FPGA.

Resource	LUT (47,232)	FF (94,464)	BRAM (150)	DSP (240)	f/MHz
System consumption	41,369 (87.6%)	47,111 (49.9%)	96 (64.0%)	223 (92.9%)	100
Convolution consumption	23,361 (49.5%)	21,878 (23.2%)	92 (61.3%)	199 (82.9%)	100

**Table 2:** Comparison of the different solutions.

Parameters	CPU	Paper [37]	Paper [38]	Paper [39]	This Paper
Experimental Platform	Core i5-10210U	Zedboard	Zedboard	Stratix V GSD8	AXU2CGB
Quantization	Float-32	Fixed-16	Fixed-16	Fixed-16	Fixed-16
Frequency/Hz	1.6 G	100 M	100 M	120 M	100 M
Inference time per img/s	1.751	18.025	0.532	0.651	0.468
Mean average precision (mAP)	90.76%	69%	30.9%	87.48%	89.33%
Power/W	15.25	2.384	3.36	25.40	3.52

## Data Availability

The datasets generated during and/or analyzed during the current study are available from the corresponding author on reasonable request.

## Abbreviations

The following abbreviations are used in this manuscript:

mAP	mean average precision
CNN	convolutional neural network
FPN	feature pyramid network
BN	batch normalization
AP	average precision
TP	ture positives
FP	false positives
FN	false negatives
BRAM	block RAM
MIO	multiuse I/O
PEs	processing elements
TDM	time division multiplexing
IoT	internet of things
